# Practical approach to COVID-19: an Egyptian pediatric consensus

**DOI:** 10.1186/s43054-020-00037-9

**Published:** 2020-08-28

**Authors:** Abla S. Mostafa, Ashraf Abdalbaky, E. M. Fouda, Hala H. Shaaban, Hala G. Elnady, Magda Hassab-Allah, Mohamed M. Rashad, Mona M. El Attar, Mostafa Alfishawy, Shahenaz M. Hussien, Tarek Hamed, Dina H. Hamed, Dina T. Sarhan

**Affiliations:** 1 Pediatric Pulmonology, Faculty of Medicine, Cairo University, Giza, Egypt; 2 Pediatrics, Faculty of Medicine, Ain-Shams University, Cairo, Egypt; 3 Pediatric Pulmonology, Faculty of Medicine, Ain-Shams University, Cairo, Egypt; 4 Pediatric Pulmonology and Allergy, Faculty of Medicine, Cairo University, Giza, Egypt; 5 Child Health, Medical Division, National Research Center, Giza, Egypt; 6 Pediatrics, Faculty of Medicine, Al-Azhar University, Cairo, Egypt; 7 Pediatrics and Pediatric Pulmonology, Faculty of Medicine, Benha University, Benha, Egypt; 8 Pediatrics, Faculty of Medicine, Cairo University, Giza, Egypt; 9 Infectious Disease, Aswan Heart Center, Aswan, Egypt; 10 Pediatrics, Faculty of Medicine, Al-Azher University, Cairo, Egypt; 11 Pediatric Department, Faculty of Medicine, Zagazig University, Zagazig, Egypt; 12 Department of Pediatrics, Faculty of Medicine, University Hospital for Children, Zagazig University, Zagazig City, Al-Sharkiya Governorate Egypt

**Keywords:** COVID-19, Pandemic, Egyptian, Health care worker, Breastfeeding, Pediatrics

## Abstract

**Background:**

Outbreak of a novel corona virus was reported in China on December 2019. Sooner, a global spread was reported and WHO announced a public health emergency of international concern and then declared it as a pandemic. Egypt announced the first case on February 14, 2020, and since that time, cases are increasing.

**Main body:**

There is increasing need to simplify the practical approach for pediatricians and other health care workers in a step wise manner; how to deal with COVID-19 cases, how to care for the newborn babies as regards to breastfeeding, and how to ensure safety of health care workers assess their risk of infection and management accordingly. A national practical approach guideline was prepared including case definition, diagnosis, and management of pediatric COVID-19 suspected and confirmed cases in an algorithmic pattern.

**Conclusion:**

Up to the current knowledge, this is a simple and practical guidance for clinical management of children during the current pandemic.

## Background

A novel coronavirus was identified following a cluster of cases of pneumonia in Wuhan, China, in December 2019 [1]. It rapidly spread as an outbreak there. A limited human to human transmission mainly within families was recorded, and the World Health Organization (WHO) announced this on January 22, 2020. On the 23rd of January, it was announced that the outbreak constituted a public health emergency of international concern [2].

WHO designated the disease as coronavirus disease 2019 (COVID-19) and the causative agent severe acute respiratory syndrome coronavirus 2 (SARS-CoV-2) in February 2020 [3]. Few weeks later, virus spread was recorded worldwide and was announced as a pandemic by WHO in March 11, 2020 [4].

Global spread included Egypt, and the first case was recorded in Egypt on February 14, 2020 [5]. The total number of confirmed cases on May 1, 2020, was 5895, with case fatality rate of 6.9%. Children were affected like other age groups, but total incidence was less than 10%. Confirmed cases among health care workers were 11% of the total confirmed cases [6].

## Main text

Egypt is one of the lower-middle-income countries with limited resources [7] which require a simple and practical clinical guideline to diagnose and treat COVID-19 cases, as well as to protect health care workers from catching infection.

Breastfeeding by COVID-19 mother is another problem that has to be addressed. So, we found a need to advocate this algorithmic approach to simplify these aims.

The science is evolving rapidly and liable to change, and up to the current knowledge, this is a simple and practical guidance for clinical management of children during the current pandemic.

## Methodology

This consensus statement is based on expert opinions of pediatric pulmonologists and infectious diseases consultant representing different universities all over Egypt. They are current active members of the Egyptian Pediatric Clinical Practice Guidelines Committee (EPG); several members of the panel have experience with managing pediatric patients during the current pandemic. The panel met via multiple live audio and video conference calls to discuss the most recent international guidelines, data, and recommendations until consensus was achieved [8–12].

The final document is supported by an extensive literature review utilizing the search terms (COVID-19 or SARS-CoV2 or Coronavirus 2019 and pediatrics or children).

## Discussion

To minimize the risk of transmission of infection to healthcare workers, these precautions are taken.

### In triage (emergency room)

The child suspected of COVID-19 is transferred to a specialized triage room which is a separate room with good aeration located outside the emergency room (ER)—isolated compartments—prior to any waiting area, better if there is available negative pressure room in the ER. The room contains ready to use soap and hand washing station or alcohol-based hand rub.

The child and his parents wear surgical masks (if tolerated).

All the triage health care workers (HCW) are also instructed to have at least surgical mask, minimize the duration of exposure, and keep safe distance 1–2 m as possible to decrease risk of infection [8].

The examiner doctor and triage personnel who will be taking vitals and assessing patients also must follow the standard and droplet precautions during the examination by wearing their personal protective equipment (PPE) including a respirator (or facemask if respirators are not available), eye protection, and gloves for the primary evaluation of all patients presenting for care until COVID-19 is proved unlikely [13].

### Inside the hospital

As community transmission intensifies within our region, HCW should wear a facemask all times while they are in the healthcare facility. The burden of care lies in the management of suspected cases in the initial 48-h period prior to the swab results.

Home care is preferable if the child’s situation and house condition allows.

When admitted, the patient with known or suspected COVID-19 is placed in a single-person room with the door closed and possible a dedicated bathroom. In the absence of a private room, patients are housed in the same room with at least two meters away in between. Each child can only be accompanied by one caregiver who is provided with a surgical facemask and advised to stay in the room with the child at all times [14].

HCW who enter the room of a patient with known or suspected COVID-19 should adhere to standard precautions and use a respirator (or facemask if a respirator is not available), gown, gloves, and eye protection with proper hand hygiene [8].

Whenever possible, procedures/tests are performed in the patient’s room; nebulization is limited due to the risk of aerosol droplets infection. Instead, metered dose inhalers are preferred [15]. When other aerosol generating procedures are performed, the number of HCW present during the procedure is limited to only those essential for patient care and procedure support, wearing an N95 respirator, eye protection, overheads, gloves, and a gown. The procedure is followed by cleaning and disinfecting measures for room surfaces promptly [16].

These precautions are to ensure safety of HCW, assess their risk of infection, and manage accordingly (Algorithm 1).

**Algorithm 1:**
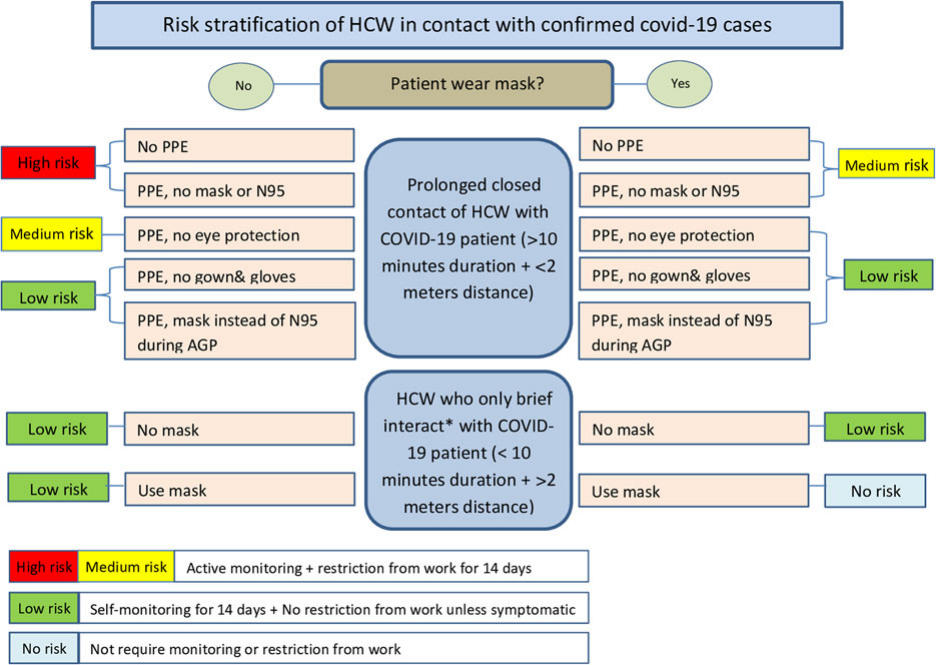
Risk stratification of HCW in contact with confirmed COVID-19 cases. Abbreviations: *HCW* health care worker, *AGP* aerosol generating procedure, *PPE* personal protective equipment. *Brief interactions include: brief conversation at a triage desk; briefly entering a patient room but not having direct contact with the patient or the patient’s secretions/excretions; entering the patient room immediately after the patient was discharged. A record of HCW exposed to suspected COVID-19 patient should be maintained and HCW should be encouraged to perform self-monitoring while awaiting test results. If the results will be delayed more than 72 hours or the patient proved positive for COVID-19, then the monitoring and work restrictions described in this algorithm should be followed

### Diagnosis of COVID-19 cases

Diagnosis of COVID-19 will depend on a case definition of suspected and confirmed case (Algorithm 2). Implementation of case definition will depend on the clinical presentation of the case and on laboratory test as well as radiological finding. The cases will be stratified according to these collective data to different grades of severity [17].

**Algorithm 2:**
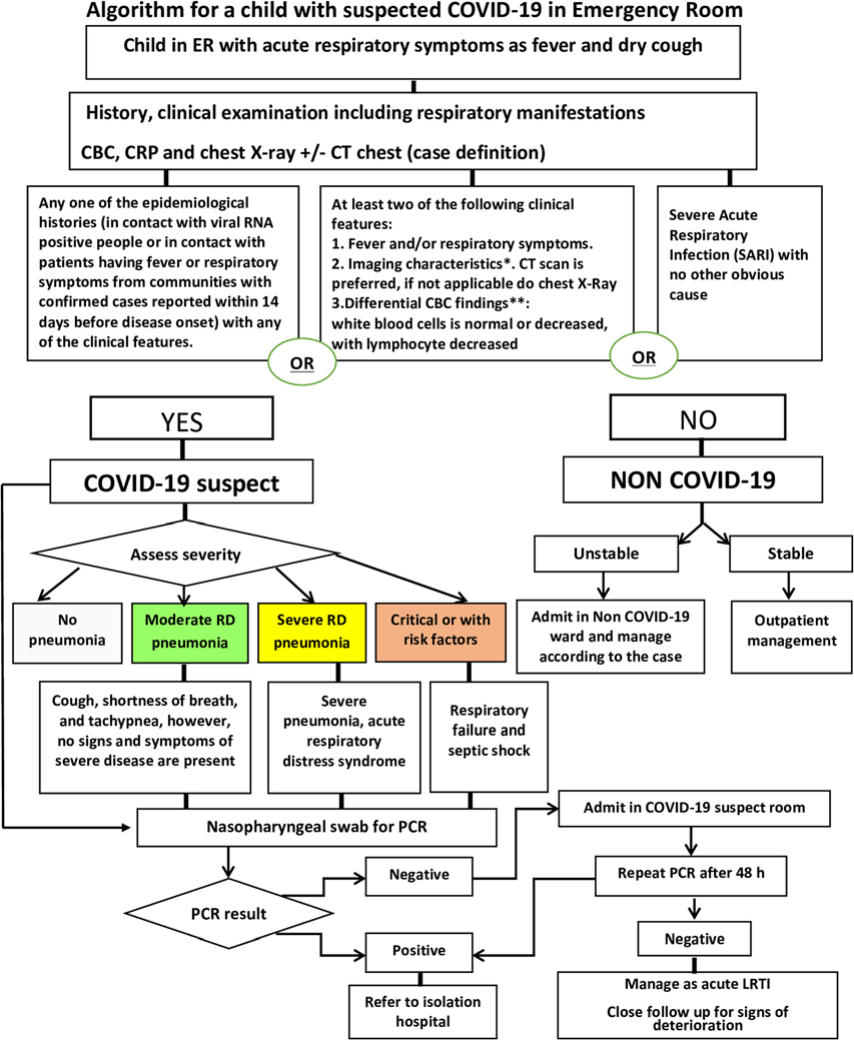
Algorithm for a child with suspected COVID2 Algorithmemergency room. Abbreviations: *RD* Respiratory distress, *PCR* Polymerase chain reaction, *LRTI* Lower respiratory tract infection

Mild cases are either asymptomatic or symptomatic with Leucopenia and/or lymphopenia with no radiological evidence of pneumonia (upper respiratory tract illness ± one of the following symptoms: fever < 38, cough, GIT symptoms, myalgia and/or arthralgia).

Moderate cases include patients with leucopenia and/or lymphopenia with clinical and radiological evidence of pneumonia, including fever > 38 °C with or without cough and tachypnea (respiratory rate > 60 breaths/min for infants < 2 months, > 50 breaths/min for infants 2–12 months, > 40 breaths/min for children 1–4 years, > 30 breaths/min for children older than 5 years old), and the condition may be associated by moderate to severe dehydration.

Cases are considered as severe and critically ill if any of the following is present:

- O_2_ saturation ≤ 92% or Pa O2/FiO2 < 200 despite escalating O2 therapy to maximal allowed 6 L/min
- O_2_ saturation ≤ 90 % or Pa O2/FiO2 < 300 at room air
- If the patient in septic shock or confused or hemodynamically unstable despite fluid resuscitation
- If respiratory manifestations are combined with other organ failure
- Chest radiography > 50% lesion or progressive lesion within 24–48 h

### Investigations

For suspected case:

CBC and radiology

To confirm diagnosis:

Nasopharyngeal swab for PCR

To assess severity:

Serum ferritin, D dimer, LDH, and CRP

### Chest radiological abnormalities

- *Chest x-ray abnormalities*:
  - Bilateral lung infiltrates are found in 75% of patients, and unilateral lung infiltrates are found in 25% of patients.
- *Abnormalities in C T chest*:
  - Bilateral multiple lobular and sub segmental areas of ground-glass opacity or consolidation and/or reverse halo sign
  - Usually with a peripheral or posterior distribution, mainly in the lower lobes [18].

### Laboratory abnormalities

- CBC: WBCs may be normal or low or high (lymphocytopenia is present in > 80% in patients). Other findings may include thrombocytopenia and decreased hemoglobin. Neutrophilia: with neutrophil/lymphocyte ratio on CBC ≥ 3.1 is characteristic.
- Elevated liver transaminases, CRP, LDH, D-dimer, and serum ferritin.
- Decreased albumin and renal impairment [19].
- IL-6 level if available in sever critical cases.

### High-risk children

1. Any chronic disease such as diabetes, kidney disease, undergoing dialysis, moderate to severe asthma, serious heart conditions, liver disease, and severe obesity (BMI > 40).
2. Immunocompromising conditions including active malignancy, cancer treatment, bone marrow or organ transplantation, poorly controlled HIV or AIDS, and prolonged use of corticosteroids and other immunosuppressive drugs [20].

### Treatment

Currently, there are no Food and Drug Administration (FDA)-approved drugs for COVID-19. Neither the World Health Organization nor the US Centers for Disease Control and Prevention recommends any specific anti-COVID-19 treatment in children [9, 10].

However, an array of drugs approved for other indications, as well as multiple investigational agents, are being studied for treatment and prevention or post-exposure prophylaxis are under way in several hundred clinical trials around the globe [21], but evidence on effective treatments is not yet available; thus, use of specific drugs should be under medical and regulatory supervision to establish safety and efficacy. Current treatment for COVID-19 is mainly supportive care [22].

The decision of the site of management either at home or in hospital depends on the clinical presentation, requirement for supportive care, potential risk factors for severe disease, and the ability of the patient to self-isolate at home [10, 23].

Supportive treatment including sufficient fluid and calories intake, and additional oxygen supplementation should be used in the treatment of children infected with COVID-19. The aim is to prevent ARDS, organ failure, and secondary nosocomial infections. If bacterial infection is suspected, broad-spectrum antibiotics may be used [24].

To make the practical management easy and applicable with the current Egyptian limited resources and available medical facilities, the key recommendations for treating pediatric COVID-19 patients according to severity of disease are summarized in Table 1 and Algorithm 3, pediatric drug doses of used medications are listed in Table 2, with special guide to the indications, contraindications, and pediatric dosing of anticoagulants which are listed in Table 3 (a, b); also, the discharge standards and follow-up plan are illustrated in Table 4.

**Table 1:** Key recommendations for treating pediatric COVID-19 patients according to severity of disease

Treatment Diagnosis	Reassurance	IPC & Contact tracing	Symptomatic treatment	Antibiotics	Antivirals	Immunomodulatory	Anticoagulants
Asymptomatic	+	+	-	-	-	-	-
Mild	+	+	+	-	-	-	-
Moderate	+	+	+	+	+	+/-	Prophylactic or Therapeutic
Severe	+	+	+	+	+	+/-	Therapeutic

*IPC* infection prevention and control

**Algorithm 3:**
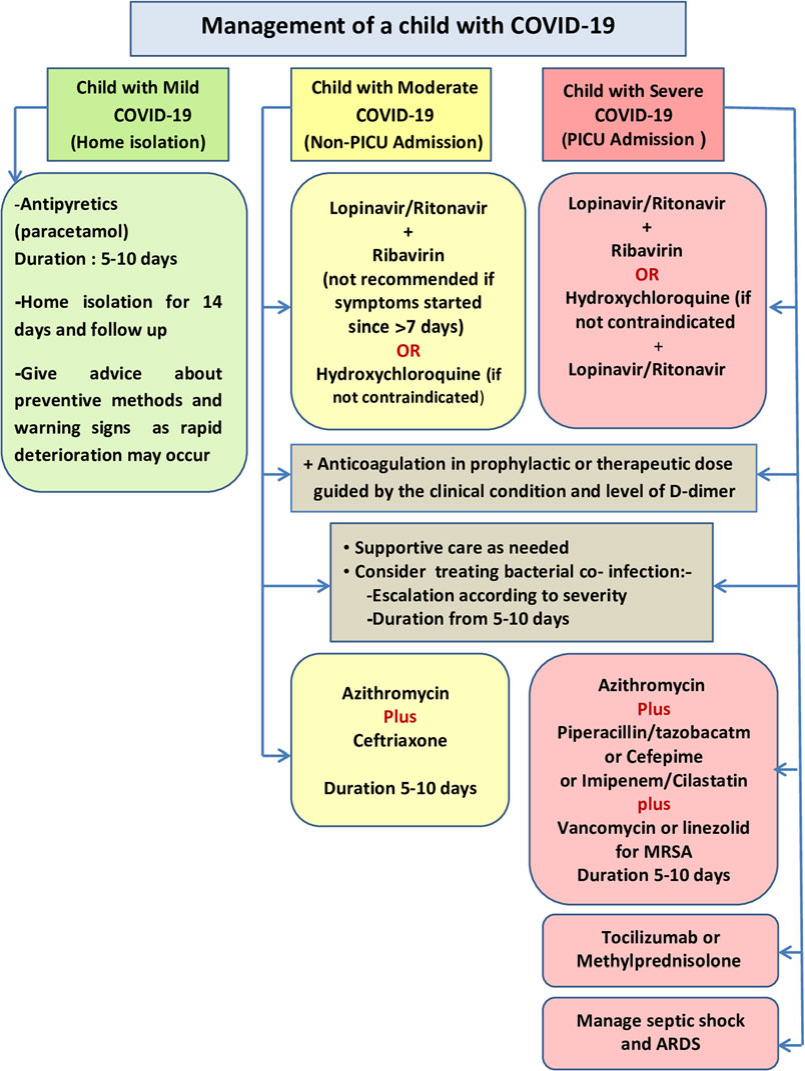
Management of a child with COVID-19. Abbreviations: *PICU* Pediatric intensive care unit, *MERSA* Methicillin-resistant Staphylococcus aureus, *ARDS* Acute respiratory distress syndrome

**Table 2:** Pediatric drug doses of used medications

Drug	Pediatric Dose	Notes
Azithromycin	10 mg/kg once on day 1 (maximum dose: 500 mg/dose) , followed by 5 mg/kg (maximum dose: 250 mg/dose) once daily on days 2 to 5	• Monitor ECG in high risk patients due to the risk of QTc prolongation. • For 5 days
Ceftriaxone	100 mg/kg/dose once daily (maximum daily dose: 2gm mg/day)	• For 5-10 days
Ribavirine	children> 3 years of age: 15 mg/kg /day in 2 divided oral doses	• Patients with impaired renal function, adjust ribavirine dose according to nephrologist advice.
		• Monitor CBC, serum creatinine, liver function
		• For 14 days
Lopinavir/ Ritonavir	Dosage based on weight, presented based on mg of lopinavir; maximum dose: Lopinavir 400 mg/ritonavir 100 mg 7–15 kg: 12 mg/kg twice daily 15–40 kg: 10 mg/kg twice daily >40 kg: 400 mg/100 mg twice daily	• Do not use lopinavir/ ritonavir in pre-term or full term neonates before 14 days of age.
		• Check for drug-drug interaction (consult clinical pharmacist).
		• If lopinavir/ritonavir is not available may consider darunavir/cobicistat as an alternative.
		• For 14 days
Hydroxychloroquine	6.5 mg/kg orally every 12 hours (max: 600 mg/dose) for two doses, followed by 3 mg/kg orally every 12 hours (max: 200 mg/dose) for a total of 5-10 days	• Check contraindications carefully
	Contraindication :	• Use with caution in QT interval prolongation
	Allergy to 4-aminoquinoline	• Pediatric dose may change based on future studies
	Chronic liver and kidney disease or Hematological Disorders	• For 5-10 days
	Patients with arrhythmia and chronic heart disease	
	Patients known to have retinal disease or hearing loss	
	Skin disorders (including rash, dermatitis, and psoriasis)	
	Glucose-6-phosphate dehydrogenase (G6PD) deficiency	
	Revise drug drug interaction carefully with pharmacist	
Piperacillin/Tazobactam	300 mg/ kg/day divided every 6-8 hours	• For 5-10 days but may be extended on a case-bycase basis
Vancomycin	15 mg/kg/dose every 6 hours	• For 5-10 days but may be extended on a case-bycase basis
Tocilizumab	<30 kg: 12 mg/kg	• H score with a value more than 169
	≥30 kg: 8 mg/kg (max: 800 mg/dose)	• Duration: One dose
		• Response usually seen at 48-72h
		• Don't exceed 800 mg/dose
Methylprednisolone	2 mg/kg/day	• For 3-5 days when Tocilizumab not available

**Table 3:** Pediatric COVID-19 anticoagulation regimens

**a. Doses of anticoagulation**		
	Prophylaxis	Therapeutic dose
Heparin	100–150 units/kg IV once	*Infusion* • < 1 year—loading dose of 75 units/kg IV, then 28 units/kg/h IV as initial maintenance dose • 1 year—Loading dose of 75 units/kg IV, then 20 units/kg/h IV as initial maintenance dose *Intermittent IV injection* • Initially give 50–100 units/kg IV infusion, then 100 units/kg IV infusion q4hr as a maintenance dose
Enoxaparin	< 2 months: 0.75 mg/kg SC q12h ≥ 2 months: 0.5 mg/kg SC q12hr	< 2 months: 1.5 mg/kg SC q12h ≥ 2 months: 1 mg/kg SC q12hr
**b. Indications and contraindications of anticoagulation**		
Indications for prophylactic anticoagulation		All admitted patients with moderate COVID-19, i.e., any patient with pneumonia All moderate and severe cases who don't meet the therapeutic indications
Indications for therapeutic anticoagulation		All ICU patients O2 sat < 92% or tachypnea for age O2 requirements ≥ 4 L on nasal cannula Elevated D-dimer 3x upper level of normal Elevated CRP
Contraindications		Patients who are at high risk of bleeding Platelets < 50,000 INR > 1.5 Active bleeding

**Table 4:** Discharge standards and follow-up plan

Discharge standards	Medication after discharge	Follow-up plan
**1-** Body temperature: normal for > 3 days. **2-** Respiratory symptoms: significantly improved. **3-** PCR: negative for respiratory tract pathogen twice (48 h apart). **4-** Lung imaging: showing obvious improvement. **5-** No comorbidities or complications which require hospitalization. **6-** SpO_**2**_, > 94% without assisted oxygen inhalation. **7-** Discharge approved by multi-disciplinary medical team.	***Symptomatic Treatment*:** as needed ***Antiviral drugs***: for patients with multiple lung lesions in the first 3 days after their nucleic acid tests are negative.	***Follow-up call***: within 48 h after discharge. ***Outpatient follow-up***: 1 week, 2 weeks after discharge. ***Examinations***: include • CBC&CRP • Lung CT scan according to the patient's condition. • PCR test of sputum and stool samples. • Liver and kidney functions, ***Follow-up phone calls***: 3 and 6 months after discharge.

### Feeding the newborn of COVID-19 mother

As breast milk samples from the COVID-19–positive mothers after the first lactation were all negative for the virus, and as breastfeeding is particularly effective against infectious diseases because it strengthens the immune system by several mechanisms including direct transferring of antibodies from the mother, and other anti-infective factors and long-lasting transfer of immunological competence and memory; therefore, all confirmed or suspected COVID-19 mothers with any symptoms who are breastfeeding or practicing skin-to-skin contact should follow standard infant feeding guidelines with appropriate precautions [9, 25] (Algorithm 4).

**Algorithm 4:**
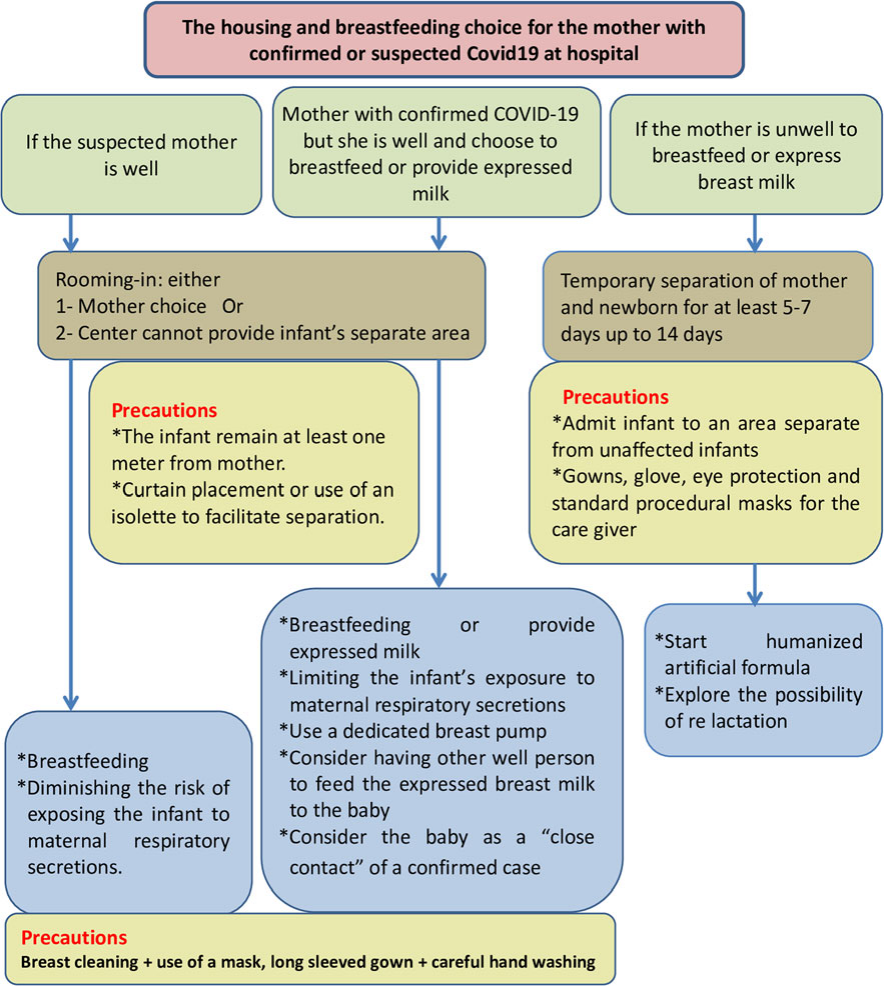
The housing and breastfeeding chioce for the mother with confirmed or suspected COVID-19 at hospital

## Conclusion

Covid-19 is nowadays a community-acquired infection. HCW must wear all recommended PPE to protect themselves from getting infected. Especial care must be considered for newborn baby and to continue as much as possible breast feeding. Diagnosis and management of suspected COVID-19 children must be updated to latest international experience in this field, and implementation of a national guideline is important.

It is important to stress that the treatment recommendations in this paper should not be considered mandates. The choice of what to do or not to do for an individual patient is ultimately decided by the patients together with their health care providers.

## Data Availability

Authors can confirm that all relevant data are included in the article and/or its supplementary information files.

## Abbreviations

AIDS: Acquired immunodeficiency syndrome
ARDS: Acute respiratory distress syndrome
CBC: Complete blood count
COVID-19: Coronavirus disease 2019
CRP: C-reactive protein
EPG: Egyptian Pediatric Clinical Practice Guidelines Committee
ER: Emergency room
FDA: Food and drug administration
HCW: Health care workers
HIV: Human immunodeficiency virus
LDH: Lactate dehydrogenase
PCR: Polymerase chain reaction
PPE: Personal protective equipment
WHO: World Health Organization
